# 3-Benzyl­amino-2-cyano-*N*-[*N*-(2-fluoro­phenyl)­carbamo­yl]-3-(methyl­sulfanyl)acryl­amide

**DOI:** 10.1107/S1600536811046125

**Published:** 2011-11-09

**Authors:** Shihua Zhong, Mingliang Fan, Jianbing Liu, Bingyu Liu

**Affiliations:** a College of Chemistry and Chemical Engineering, Hunan Normal University, Changsha 410081, Hunan, People’s Republic of China

## Abstract

In the crystal structure of the title compound, C_19_H_17_FN_4_O_2_S, mol­ecules are linked *via* pairs of N—H⋯N inter­actions, forming centrosymmetric dimers. Two intra­molecular N—H⋯O hydrogen bonds stabilize the mol­ecular conformation.

## Related literature

The title compound was synthesized as a herbicide. For details of the synthesis, see: Wang *et al.* (2004 ▶); Senda *et al.* (1972 ▶); Xue *et al.* (2002 ▶); Liu *et al.* (1998 ▶); Zhang *et al.* (2008 ▶).
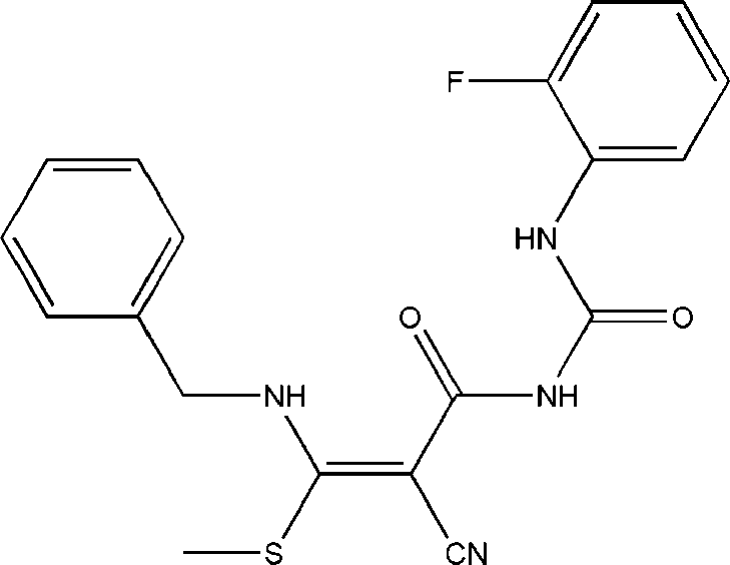


## Experimental

### 

#### Crystal data

C_19_H_17_FN_4_O_2_S
*M* *_r_* = 384.43
Triclinic, 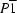

*a* = 9.2415 (18) Å
*b* = 10.047 (2) Å
*c* = 11.0949 (19) Å
α = 73.312 (6)°
β = 66.880 (6)°
γ = 84.295 (7)°
*V* = 907.4 (3) Å^3^
*Z* = 2
Mo *K*α radiation
μ = 0.21 mm^−1^
*T* = 133 K
0.48 × 0.39 × 0.39 mm

#### Data collection

Rigaku AFC10/Saturn724+ diffractometer
Absorption correction: multi-scan (*ABSCOR*; Higashi, 1995 ▶) *T*_min_ = 0.906, *T*_max_ = 0.923
8644 measured reflections
4089 independent reflections
3137 reflections with *I* > 2σ(*I*)
*R*_int_ = 0.025

#### Refinement

*R*[*F* ^2^ > 2σ(*F* ^2^)] = 0.035
*wR*(*F* ^2^) = 0.079
*S* = 1.00
4089 reflections
251 parameters
H atoms treated by a mixture of independent and constrained refinement
Δρ_max_ = 0.32 e Å^−3^
Δρ_min_ = −0.23 e Å^−3^

### 

Data collection: *CrystalClear* (Rigaku, 2008 ▶); cell refinement: *CrystalClear*; data reduction: *CrystalClear*; program(s) used to solve structure: *SHELXS97* (Sheldrick, 2008 ▶); program(s) used to refine structure: *SHELXL97* (Sheldrick, 2008 ▶); molecular graphics: *SHELXTL* (Sheldrick, 2008 ▶); software used to prepare material for publication: *SHELXL97*.

## Supplementary Material

Crystal structure: contains datablock(s) I, global. DOI: 10.1107/S1600536811046125/ds2144sup1.cif

Structure factors: contains datablock(s) I. DOI: 10.1107/S1600536811046125/ds2144Isup2.hkl

Supplementary material file. DOI: 10.1107/S1600536811046125/ds2144Isup3.cml

Additional supplementary materials:  crystallographic information; 3D view; checkCIF report

## Figures and Tables

**Table 1:** Hydrogen-bond geometry (Å, °)

*D*—H⋯*A*	*D*—H	H⋯*A*	*D*⋯*A*	*D*—H⋯*A*
N1—H1*N*⋯O1	0.860 (17)	1.894 (17)	2.6024 (15)	138.7 (15)
N3—H3*N*⋯O1	0.827 (16)	1.912 (16)	2.6012 (16)	140.1 (14)
N2—H2*N*⋯N4^i^	0.858 (15)	2.229 (16)	3.0710 (17)	166.8 (14)

Symmetry code: (i) 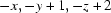.

**Fig. 1.**
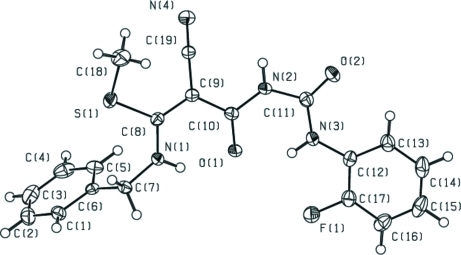
The molecular structure of compound shows displacement ellipsoids drawn at the 50% probability level, all H atoms have been omitted for clarity.

**Fig. 2.**
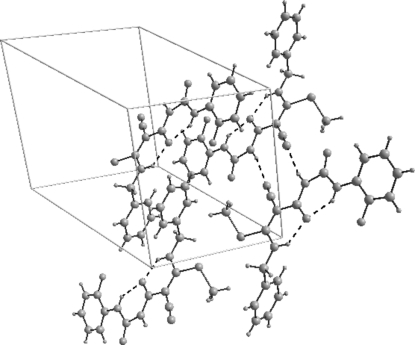
The crystal packing of the title compound with hydrogen bonds drawn as dashed lines.

## supplementary crystallographic information

### Experimental

The title compound was prepared according to the reported method (Wang *et al.*, 2004; Senda *et al.*, 1972). Crystals of (I) suitable for XRD were obtained by slow evaporation of the acetone solution at 293 K.

### Refinement

Positional parameters of all H atoms were calculated geometically and were allowed to ride on the C, O and N atoms to which they are bonded, with C—H = 0.95 to 0.99 Å and *U*_iso_(H) = 1.2*U*_eq_(C).

### Crystal data

**Table:**

C_19_H_17_FN_4_O_2_S	*Z* = 2
*M**_r_* = 384.43	*F*(000) = 400
Triclinic, *P*1	*D*_x_ = 1.407 Mg m^−^^3^
Hall symbol: -P 1	Mo *K*α radiation, λ = 0.71073 Å
*a* = 9.2415 (18) Å	Cell parameters from 2908 reflections
*b* = 10.047 (2) Å	θ = 2.4–29.1°
*c* = 11.0949 (19) Å	µ = 0.21 mm^−^^1^
α = 73.312 (6)°	*T* = 133 K
β = 66.880 (6)°	Block, colourless
γ = 84.295 (7)°	0.48 × 0.39 × 0.39 mm
*V* = 907.4 (3) Å^3^	

### Data collection

**Table:**

Rigaku AFC10/Saturn724+ diffractometer	4089 independent reflections
Radiation source: Rotating Anode	3137 reflections with *I* > 2σ(*I*)
graphite	*R*_int_ = 0.025
Detector resolution: 28.5714 pixels mm^-1^	θ_max_ = 27.5°, θ_min_ = 2.1°
phi and ω scans	*h* = −11→9
Absorption correction: multi-scan (*ABSCOR*; Higashi, 1995)	*k* = −13→13
*T*_min_ = 0.906, *T*_max_ = 0.923	*l* = −14→12
8644 measured reflections	

### Refinement

**Table:**

Refinement on *F*^2^	Primary atom site location: structure-invariant direct methods
Least-squares matrix: full	Secondary atom site location: difference Fourier map
*R*[*F*^2^ > 2σ(*F*^2^)] = 0.035	Hydrogen site location: inferred from neighbouring sites
*wR*(*F*^2^) = 0.079	H atoms treated by a mixture of independent and constrained refinement
*S* = 1.00	*w* = 1/[σ^2^(*F*_o_^2^) + (0.0296*P*)^2^ + 0.119*P*] where *P* = (*F*_o_^2^ + 2*F*_c_^2^)/3
4089 reflections	(Δ/σ)_max_ = 0.001
251 parameters	Δρ_max_ = 0.32 e Å^−^^3^
0 restraints	Δρ_min_ = −0.23 e Å^−^^3^

### Special details

**Table:**

Geometry. All e.s.d.'s (except the e.s.d. in the dihedral angle between two l.s. planes) are estimated using the full covariance matrix. The cell e.s.d.'s are taken into account individually in the estimation of e.s.d.'s in distances, angles and torsion angles; correlations between e.s.d.'s in cell parameters are only used when they are defined by crystal symmetry. An approximate (isotropic) treatment of cell e.s.d.'s is used for estimating e.s.d.'s involving l.s. planes.
Refinement. Refinement of *F*^2^ against ALL reflections. The weighted *R*-*wR* and goodness of fit *S* are based on *F*^2^, conventional *R*-factors *R* are based on *F*, with *F* set to zero for negative *F*^2^. The threshold expression of *F*^2^ > σ(*F*^2^) is used only for calculating *R*-factors(gt) *etc*. and is not relevant to the choice of reflections for refinement. *R*-factors based on *F*^2^ are statistically about twice as large as those based on *F*, and *R*- factors based on ALL data will be even larger.

### Fractional atomic coordinates and isotropic or equivalent isotropic displacement parameters (Å^2^)

**Table:**

	*x*	*y*	*z*	*U*_iso_*/*U*_eq_	
S1	0.42534 (5)	0.14869 (4)	0.89857 (4)	0.02801 (11)	
F1	0.21687 (11)	0.56288 (9)	0.25643 (8)	0.0357 (2)	
O1	0.27259 (13)	0.42794 (11)	0.56187 (9)	0.0295 (3)	
O2	−0.00916 (14)	0.75621 (12)	0.64645 (10)	0.0363 (3)	
N1	0.43164 (14)	0.22266 (12)	0.65160 (11)	0.0222 (3)	
N2	0.10887 (14)	0.55174 (12)	0.70402 (12)	0.0228 (3)	
N3	0.11794 (15)	0.64303 (13)	0.48270 (12)	0.0239 (3)	
N4	0.05563 (16)	0.35724 (14)	1.03305 (12)	0.0341 (3)	
C1	0.63303 (18)	−0.12037 (16)	0.63660 (14)	0.0265 (3)	
H1	0.7399	−0.0901	0.5950	0.032*	
C2	0.5979 (2)	−0.26010 (17)	0.67324 (16)	0.0360 (4)	
H2	0.6802	−0.3254	0.6567	0.043*	
C3	0.4422 (2)	−0.30499 (17)	0.73435 (16)	0.0394 (4)	
H3	0.4175	−0.4012	0.7596	0.047*	
C4	0.3231 (2)	−0.20958 (17)	0.75847 (15)	0.0347 (4)	
H4	0.2164	−0.2403	0.8006	0.042*	
C5	0.35910 (18)	−0.06872 (16)	0.72123 (14)	0.0259 (3)	
H5	0.2768	−0.0035	0.7382	0.031*	
C6	0.51439 (16)	−0.02309 (14)	0.65956 (12)	0.0199 (3)	
C7	0.56151 (16)	0.12821 (14)	0.61482 (14)	0.0223 (3)	
H7A	0.6365	0.1390	0.6553	0.027*	
H7B	0.6175	0.1559	0.5146	0.027*	
C8	0.36183 (16)	0.25079 (14)	0.77143 (13)	0.0194 (3)	
C9	0.24408 (17)	0.35141 (14)	0.79187 (13)	0.0219 (2)	
C10	0.21072 (17)	0.44413 (15)	0.67827 (13)	0.0218 (3)	
C11	0.06720 (17)	0.66004 (15)	0.60914 (14)	0.0235 (3)	
C12	0.10494 (16)	0.73778 (15)	0.36651 (13)	0.0220 (3)	
C13	0.05148 (17)	0.87350 (16)	0.35728 (15)	0.0287 (3)	
H13	0.0137	0.9076	0.4355	0.034*	
C14	0.05355 (18)	0.95894 (17)	0.23327 (16)	0.0354 (4)	
H14	0.0189	1.0520	0.2271	0.042*	
C15	0.10502 (19)	0.91076 (18)	0.11951 (16)	0.0378 (4)	
H15	0.1044	0.9701	0.0359	0.045*	
C16	0.15778 (19)	0.77617 (18)	0.12629 (15)	0.0330 (4)	
H16	0.1927	0.7414	0.0485	0.040*	
C17	0.15823 (17)	0.69412 (15)	0.24895 (14)	0.0259 (3)	
C18	0.4107 (2)	0.25546 (18)	1.00866 (16)	0.0385 (4)	
H18A	0.4372	0.3521	0.9544	0.058*	
H18B	0.4839	0.2222	1.0548	0.058*	
H18C	0.3030	0.2501	1.0767	0.058*	
C19	0.14271 (17)	0.35427 (15)	0.92622 (13)	0.0219 (2)	
H3N	0.1721 (19)	0.5738 (17)	0.4704 (15)	0.030 (5)*	
H2N	0.0681 (19)	0.5644 (16)	0.7839 (16)	0.035 (5)*	
H1N	0.394 (2)	0.2677 (18)	0.5923 (17)	0.040 (5)*	

### Atomic displacement parameters (Å^2^)

**Table:**

	*U*^11^	*U*^22^	*U*^33^	*U*^12^	*U*^13^	*U*^23^
S1	0.0371 (2)	0.0269 (2)	0.02340 (19)	0.00577 (17)	−0.01710 (17)	−0.00569 (15)
F1	0.0496 (6)	0.0324 (5)	0.0293 (5)	0.0090 (4)	−0.0187 (4)	−0.0121 (4)
O1	0.0418 (7)	0.0305 (6)	0.0189 (5)	0.0157 (5)	−0.0141 (5)	−0.0122 (4)
O2	0.0447 (7)	0.0339 (7)	0.0331 (6)	0.0205 (6)	−0.0174 (5)	−0.0163 (5)
N1	0.0279 (7)	0.0215 (7)	0.0191 (6)	0.0080 (5)	−0.0107 (5)	−0.0082 (5)
N2	0.0267 (7)	0.0248 (7)	0.0179 (6)	0.0071 (5)	−0.0086 (5)	−0.0096 (5)
N3	0.0289 (7)	0.0202 (7)	0.0236 (6)	0.0069 (6)	−0.0117 (5)	−0.0073 (5)
N4	0.0354 (8)	0.0392 (8)	0.0242 (7)	0.0105 (7)	−0.0087 (6)	−0.0108 (6)
C1	0.0287 (8)	0.0298 (8)	0.0253 (7)	0.0082 (7)	−0.0129 (7)	−0.0125 (6)
C2	0.0572 (12)	0.0292 (9)	0.0354 (9)	0.0155 (8)	−0.0299 (9)	−0.0166 (7)
C3	0.0736 (14)	0.0225 (9)	0.0310 (8)	−0.0057 (9)	−0.0287 (9)	−0.0059 (7)
C4	0.0418 (10)	0.0380 (10)	0.0239 (8)	−0.0138 (8)	−0.0074 (7)	−0.0103 (7)
C5	0.0257 (8)	0.0303 (8)	0.0232 (7)	0.0001 (7)	−0.0070 (6)	−0.0127 (6)
C6	0.0231 (7)	0.0241 (7)	0.0140 (6)	0.0031 (6)	−0.0079 (6)	−0.0075 (5)
C7	0.0190 (7)	0.0233 (8)	0.0216 (7)	0.0028 (6)	−0.0044 (6)	−0.0074 (6)
C8	0.0229 (7)	0.0182 (7)	0.0180 (6)	−0.0018 (6)	−0.0085 (6)	−0.0046 (5)
C9	0.0264 (5)	0.0216 (5)	0.0189 (5)	0.0030 (4)	−0.0093 (4)	−0.0073 (4)
C10	0.0241 (7)	0.0222 (7)	0.0206 (7)	0.0025 (6)	−0.0083 (6)	−0.0091 (6)
C11	0.0225 (8)	0.0234 (8)	0.0262 (7)	0.0028 (6)	−0.0108 (6)	−0.0078 (6)
C12	0.0182 (7)	0.0225 (8)	0.0243 (7)	−0.0019 (6)	−0.0099 (6)	−0.0016 (6)
C13	0.0227 (8)	0.0274 (8)	0.0339 (8)	0.0034 (6)	−0.0114 (7)	−0.0052 (6)
C14	0.0254 (8)	0.0298 (9)	0.0444 (9)	0.0018 (7)	−0.0160 (8)	0.0030 (7)
C15	0.0284 (9)	0.0434 (11)	0.0354 (9)	−0.0044 (8)	−0.0190 (8)	0.0098 (7)
C16	0.0303 (9)	0.0428 (10)	0.0262 (8)	−0.0024 (7)	−0.0146 (7)	−0.0034 (7)
C17	0.0242 (8)	0.0255 (8)	0.0291 (8)	0.0005 (6)	−0.0129 (7)	−0.0051 (6)
C18	0.0453 (10)	0.0519 (11)	0.0315 (8)	0.0081 (9)	−0.0240 (8)	−0.0198 (8)
C19	0.0264 (5)	0.0216 (5)	0.0189 (5)	0.0030 (4)	−0.0093 (4)	−0.0073 (4)

### Geometric parameters (Å, °)

**Table:**

S1—C8	1.7567 (13)	C4—H4	0.9500
S1—C18	1.8041 (15)	C5—C6	1.385 (2)
F1—C17	1.3674 (16)	C5—H5	0.9500
O1—C10	1.2424 (16)	C6—C7	1.5085 (19)
O2—C11	1.2115 (16)	C7—H7A	0.9900
N1—C8	1.3303 (17)	C7—H7B	0.9900
N1—C7	1.4556 (17)	C8—C9	1.4060 (18)
N1—H1N	0.860 (17)	C9—C19	1.4223 (19)
N2—C10	1.3785 (17)	C9—C10	1.4577 (18)
N2—C11	1.4204 (17)	C12—C17	1.3847 (19)
N2—H2N	0.858 (15)	C12—C13	1.393 (2)
N3—C11	1.3499 (18)	C13—C14	1.391 (2)
N3—C12	1.4059 (17)	C13—H13	0.9500
N3—H3N	0.827 (16)	C14—C15	1.374 (2)
N4—C19	1.1488 (17)	C14—H14	0.9500
C1—C2	1.379 (2)	C15—C16	1.382 (2)
C1—C6	1.3903 (19)	C15—H15	0.9500
C1—H1	0.9500	C16—C17	1.375 (2)
C2—C3	1.386 (2)	C16—H16	0.9500
C2—H2	0.9500	C18—H18A	0.9800
C3—C4	1.382 (2)	C18—H18B	0.9800
C3—H3	0.9500	C18—H18C	0.9800
C4—C5	1.391 (2)		
			
C8—S1—C18	106.50 (7)	C9—C8—S1	124.36 (10)
C8—N1—C7	126.66 (12)	C8—C9—C19	120.11 (12)
C8—N1—H1N	113.5 (11)	C8—C9—C10	121.33 (12)
C7—N1—H1N	119.8 (11)	C19—C9—C10	118.19 (12)
C10—N2—C11	127.92 (12)	O1—C10—N2	120.88 (12)
C10—N2—H2N	120.9 (11)	O1—C10—C9	121.44 (12)
C11—N2—H2N	110.9 (11)	N2—C10—C9	117.68 (12)
C11—N3—C12	127.09 (13)	O2—C11—N3	126.13 (13)
C11—N3—H3N	116.0 (11)	O2—C11—N2	119.24 (12)
C12—N3—H3N	116.4 (11)	N3—C11—N2	114.63 (12)
C2—C1—C6	121.00 (16)	C17—C12—C13	117.29 (13)
C2—C1—H1	119.5	C17—C12—N3	116.69 (13)
C6—C1—H1	119.5	C13—C12—N3	125.92 (13)
C1—C2—C3	119.78 (16)	C14—C13—C12	119.87 (14)
C1—C2—H2	120.1	C14—C13—H13	120.1
C3—C2—H2	120.1	C12—C13—H13	120.1
C4—C3—C2	119.85 (16)	C15—C14—C13	120.97 (16)
C4—C3—H3	120.1	C15—C14—H14	119.5
C2—C3—H3	120.1	C13—C14—H14	119.5
C3—C4—C5	120.16 (16)	C14—C15—C16	120.23 (14)
C3—C4—H4	119.9	C14—C15—H15	119.9
C5—C4—H4	119.9	C16—C15—H15	119.9
C6—C5—C4	120.29 (15)	C17—C16—C15	118.02 (15)
C6—C5—H5	119.9	C17—C16—H16	121.0
C4—C5—H5	119.9	C15—C16—H16	121.0
C5—C6—C1	118.91 (14)	F1—C17—C16	119.01 (13)
C5—C6—C7	122.99 (13)	F1—C17—C12	117.38 (12)
C1—C6—C7	118.09 (13)	C16—C17—C12	123.59 (14)
N1—C7—C6	114.88 (12)	S1—C18—H18A	109.5
N1—C7—H7A	108.5	S1—C18—H18B	109.5
C6—C7—H7A	108.5	H18A—C18—H18B	109.5
N1—C7—H7B	108.5	S1—C18—H18C	109.5
C6—C7—H7B	108.5	H18A—C18—H18C	109.5
H7A—C7—H7B	107.5	H18B—C18—H18C	109.5
N1—C8—C9	121.34 (12)	N4—C19—C9	177.14 (16)
N1—C8—S1	114.28 (10)		
			
C6—C1—C2—C3	−0.2 (2)	C19—C9—C10—O1	−165.04 (14)
C1—C2—C3—C4	−0.1 (2)	C8—C9—C10—N2	−171.45 (13)
C2—C3—C4—C5	0.2 (2)	C19—C9—C10—N2	15.5 (2)
C3—C4—C5—C6	0.1 (2)	C12—N3—C11—O2	6.0 (3)
C4—C5—C6—C1	−0.5 (2)	C12—N3—C11—N2	−174.67 (13)
C4—C5—C6—C7	179.10 (12)	C10—N2—C11—O2	−170.90 (14)
C2—C1—C6—C5	0.52 (19)	C10—N2—C11—N3	9.7 (2)
C2—C1—C6—C7	−179.06 (12)	C11—N3—C12—C17	−176.54 (14)
C8—N1—C7—C6	81.84 (17)	C11—N3—C12—C13	7.2 (2)
C5—C6—C7—N1	4.96 (18)	C17—C12—C13—C14	−0.1 (2)
C1—C6—C7—N1	−175.49 (12)	N3—C12—C13—C14	176.14 (14)
C7—N1—C8—C9	175.63 (13)	C12—C13—C14—C15	1.2 (2)
C7—N1—C8—S1	−6.28 (19)	C13—C14—C15—C16	−0.8 (2)
C18—S1—C8—N1	148.59 (11)	C14—C15—C16—C17	−0.6 (2)
C18—S1—C8—C9	−33.39 (15)	C15—C16—C17—F1	−176.93 (14)
N1—C8—C9—C19	163.12 (14)	C15—C16—C17—C12	1.8 (2)
S1—C8—C9—C19	−14.8 (2)	C13—C12—C17—F1	177.34 (13)
N1—C8—C9—C10	−9.8 (2)	N3—C12—C17—F1	0.7 (2)
S1—C8—C9—C10	172.35 (11)	C13—C12—C17—C16	−1.4 (2)
C11—N2—C10—O1	−4.2 (2)	N3—C12—C17—C16	−178.01 (14)
C11—N2—C10—C9	175.25 (14)	C8—C9—C19—N4	−135 (3)
C8—C9—C10—O1	8.0 (2)	C10—C9—C19—N4	38 (3)

### Hydrogen-bond geometry (Å, °)

**Table:**

*D*—H···*A*	*D*—H	H···*A*	*D*···*A*	*D*—H···*A*
N1—H1N···O1	0.860 (17)	1.894 (17)	2.6024 (15)	138.7 (15)
N3—H3N···O1	0.827 (16)	1.912 (16)	2.6012 (16)	140.1 (14)
N2—H2N···N4^i^	0.858 (15)	2.229 (16)	3.0710 (17)	166.8 (14)

Symmetry codes: (i) −*x*, −*y*+1, −*z*+2.
